# Non-linear association of the uric acid to high-density lipoprotein cholesterol ratio with non-alcoholic fatty liver disease and diabetes effect modification in Chinese adults: a secondary analysis

**DOI:** 10.3389/fmed.2025.1686975

**Published:** 2025-10-15

**Authors:** Jialing Wu, Chuhua Chen, Hongzhe Huang, Xietong Shi

**Affiliations:** ^1^Department of Gastroenterology, Jieyang People’s Hospital, Jieyang, Guangdong, China; ^2^Department of Infection, Jieyang People’s Hospital, Jieyang, Guangdong, China

**Keywords:** non-alcoholic fatty liver disease, uric acid, high-density lipoprotein cholesterol, ratio, non-linear

## Abstract

**Background:**

Global non-alcoholic fatty liver disease (NAFLD) prevalence has risen sharply, highlighting an urgent need for improved non-invasive biomarkers. The uric acid to high-density lipoprotein cholesterol ratio (UHR) integrates key metabolic pathways in NAFLD, but its precise relationship and effect modifiers remain unclear. Herein, we explored the correlation between UHR and NAFLD.

**Methods:**

This secondary analysis used data from 1,592 Chinese adults (40–70 years) undergoing health checks. NAFLD was diagnosed via ultrasound. UHR was calculated as serum uric acid (μmol/L) divided by high-density lipoprotein cholesterol ratio (HDL-C) (mmol/L). Association between UHR and NAFLD was assessed using multivariable logistic regression, restricted cubic splines (RCS) for non-linearity, and threshold analysis. Receiver Operating Characteristic analysis was utilized to assess the ability of UHR to predict the NAFLD. Subgroup analyses explored effect modification.

**Results:**

NAFLD prevalence was 61.1%. After full adjustment, each one standard deviation (SD) increase in UHR was associated with 22% higher NAFLD odds [odds ratio (OR) = 1.22, 95% confidence interval (CI): 1.03–1.46]. RCS revealed significant non-linearity (*p* < 0.001). A threshold was identified at UHR Z-score = −0.75: below this, each 1-SD increase conferred a 4.6-fold higher NAFLD risk (OR = 4.64, 95%CI: 1.57–13.68); above it, no association was found. Diabetes significantly modified the association (P-interaction = 0.028): UHR predicted NAFLD in non-diabetics (OR = 1.27, 95% CI: 1.04–1.56) but not diabetics. UHR’s predictive ability (area under the curve = 0.670) exceeded that of uric acid or HDL-C alone.

**Conclusion:**

UHR is an independent, non-linear predictor of NAFLD in Chinese adults, with a distinct risk threshold. Its association with NAFLD is significantly modified by diabetes status. UHR represents a simple, readily available biomarker potentially enhancing NAFLD risk assessment, particularly in non-diabetic individuals.

## 1 Introduction

Non-alcoholic fatty liver disease (NAFLD) has emerged as a global public health challenge, affecting approximately 25% of the world’s population and becoming the leading cause of chronic liver disease (1). In China, its prevalence is close to 30% and continues to rise in parallel with the obesity and diabetes epidemics (2, 3). NAFLD not only drives liver-related morbidity (including steatohepatitis, cirrhosis, and hepatocellular carcinoma) but also independently increases the risk of cardiovascular disease and extrahepatic malignancies (1–3). Identifying the trajectory of NAFLD is essential, as timely action in high-risk individuals could avert its onset. Preventive strategies should focus on promoting balanced nutrition, regular physical activity, and weight management (4, 5). Yet, in routine clinical practice, there remains a conspicuous lack of inexpensive, readily available biomarkers that can reliably flag individuals at heightened risk before irreversible histological damage occurs. There is thus an urgent demand for inexpensive, routinely available biomarkers to enhance risk stratification in primary care and large-scale screening.

Currently, the assessment of NAFLD risk and diagnosis relies on a spectrum of tools. Liver biopsy remains the diagnostic gold standard for assessing steatosis, inflammation, and fibrosis but is invasive and impractical for large-scale screening (6). Non-invasive imaging modalities such as transient elastography and magnetic resonance imaging-proton density fat fraction offer excellent accuracy but are cost-prohibitive and not readily available in primary care settings (6). Several biomarker panels (e.g., FIB-4) have been developed primarily for assessing fibrosis stage rather than early steatosis (7). In routine clinical practice, there remains a heavy reliance on basic measures like liver enzymes, which lack sensitivity and specificity, and components of the metabolic syndrome. This creates a conspicuous gap for an inexpensive, routinely available biomarker that can be derived from standard blood tests to reliably flag individuals at heightened risk for early NAFLD before irreversible histological damage occurs.

Uric acid (UA) and high-density lipoprotein cholesterol (HDL-C) represent two pivotal metabolic hallmarks of NAFLD pathogenesis. Elevated UA fuels hepatic lipogenesis through oxidative stress and inflammasome activation (8), while reduced HDL-C reflects impaired reverse cholesterol transport and loss of anti-inflammatory protection (9, 10). The uric acid to high-density lipoprotein cholesterol ratio (UHR) integrates these opposing pathways into a single metric, theoretically offering superior pathophysiological relevance over either component alone. Emerging evidence suggests UHR correlates with NAFLD across diverse populations (11–16); However, pivotal questions remain unanswered. Studies have largely overlooked body-composition indices such as the fat-to-muscle ratio (FMR), despite its robust association with steatosis (17). The precise configuration of the UHR–NAFLD dose–response relationship—linear, threshold, or J-shaped—has yet to be delineated. Moreover, whether diabetes or other metabolic comorbidities reshape this association remains insufficiently examined, constraining individualized risk assessment.

This study aimed to establish whether UHR served as an independent, non-linear predictor of NAFLD in Chinese adults, and to identify subpopulations most likely to benefit from UHR-based risk stratification.

## 2 Methods

### 2.1 Study population

We conducted a secondary analysis of data originally collected by Yan et al. (18). and deposited in the Dryad repository (19). The parent study received ethical approval from the Ethics Committee of Tongji Medical College, Huazhong University of Science and Technology, in accordance with the Declaration of Helsinki, and a waiver of informed consent was granted; no further ethical review was required for the present analysis because the dataset is publicly available and covered by the original ethics statement. The parent study aimed to evaluate the association between the FMR and NAFLD. Participants were adults undergoing routine health examinations at Wuhan Union Hospital between January 2020 and November 2021 and completed a standardized questionnaire on sex, age, smoking, drinking, and medical and medication history. Exclusion criteria were excessive alcohol intake (>210 g/week for male, >140 g/week for female), known liver disease (viral, autoimmune or drug-induced), acute illness, renal insufficiency (estimated glomerular filtration rate <60 mL/min/1.73 m^2^), active cancer (diagnosed or treated within the previous 6 months), current use of oral or injectable corticosteroids, or missing biochemical or questionnaire data. After applying these criteria, 1,592 participants aged 40–70 years remained for the final analyses.

### 2.2 Data collection and definitions

Anthropometric measurements were conducted by trained technicians in a dedicated hospital body composition suite. Participants fasted overnight, wore light clothing, and stood barefoot for assessments. Body composition (weight, fat mass, muscle mass) was evaluated using a multi-frequency bioelectrical impedance analyser (BIA; Tsinghua Tongfan BCA-2A, China), employing a segmented impedance measurement model. The device underwent periodic manufacturer calibration. The FMR was calculated by dividing the fat mass by the muscle mass. Height was measured, and body mass index (BMI) was calculated as weight/height^2^ (kg/m^2^). Seated blood pressure was recorded after ≥10 min of rest using an electronic sphygmomanometer (Panasonic EW3106, China), with duplicate measurements at 5-min intervals averaged for analysis.

Fasting venous blood samples (≥8 h) were processed in the hospital’s central laboratory. Platelet count (PLT) was determined using a Beckman-Coulter hematology analyser. Biochemical parameters—including total cholesterol (TC), triglycerides (TG), HDL-C, low-density lipoprotein cholesterol (LDL-C), alanine aminotransferase (ALT), aspartate aminotransferase (AST), UA, and fasting blood glucose (FBG)—were analyzed on a Beckman AU5800 automated system.

Diabetes was defined as a self-reported history of diabetes or current use of glucose-lowering medication. Hypertension was similarly defined using self-reported history or antihypertensive medication use. Fatty liver diagnosis was established through conventional abdominal B-mode ultrasound (Philips IU22, Philips Healthcare) performed by trained technicians. Participants demonstrating fatty liver without other liver comorbidities were classified as having NAFLD. The exposure variable was UHR, calculated as serum UA (μmol/L) divided by serum HDL-C (mmol/L).

### 2.3 Statistical analysis

Participants were divided into tertiles based on the UHR: tertile 1 (*n* = 530, < 227.34), tertile 2 (*n* = 532, 227.34–408.15) and tertile 3 (*n* = 530, ≥ 408.15). Categorical variables were reported as *n* (%) and compared with *χ*^2^ tests; normally distributed continuous variables were presented as mean ± standard deviation (SD) and compared by one-way ANOVA, whereas non-normally distributed variables were expressed as median (interquartile range) and compared with the Kruskal–Wallis test.

Logistic regression analyses were employed to assess the association between UHR and the risk of NAFLD, quantified by odds ratios (ORs) and 95% confidence intervals (CIs). Model 1 was unadjusted; Model 2 included sex, age, diabetes, hypertension, tobacco use and alcohol use; Model 3 further adjusted for BMI, FMR, PLT, ALT, AST, FBG, TC, TG, LDL-C, SBP and DBP. Restricted cubic splines (RCS) were used to model potential non-linear relationships between UHR and NAFLD. The Likelihood Ratio Test was applied to evaluate the non-linearity component within the RCS model. To rigorously investigate the shape of the UHR-NAFLD association—specifically, to determine linearity versus non-linearity and identify potential threshold effects—a threshold effect analysis was conducted. This analysis employed two competing models: Model 1, a standard logistic regression assuming a linear relationship between UHR and NAFLD risk; and Model 2, a two-piecewise logistic regression model fitted around a potential inflection point (threshold). The Likelihood Ratio Test was then used to adjudicate which model (the linear Model 1 or the piecewise Model 2) provided a statistically significantly better fit to the data.

Subgroup analyses were performed to examine potential effect modification by sex, age (≤ 60 vs. > 60 years), BMI (< 25 vs. ≥ 25 kg m^−2^), tobacco use, alcohol use, hypertension and diabetes. Interaction terms were tested separately.

The predictive performance of UHR for NAFLD was evaluated using receiver operating characteristic (ROC) curve analysis. The area under the ROC curve (AUC) for UHR was compared to the AUCs of its individual components, UA and HDL-C, using DeLong’s test.

All statistical analyses were performed using SPSS (version 27.0; SPSS Inc., Chicago, IL) and R software (version 4.0.5). Statistical significance was defined as a two-sided *p* value < 0.05.

## 3 Results

### 3.1 Characteristics of the population by UHR

The study included 1,592 patients (male: female ratio = 2.59) with a mean UHR of 360.83. NAFLD was diagnosed in 973 patients (61.1%). The characteristics of the patients by tertile of the UHR are shown in Table 1. Across increasing tertiles, the proportions of males and patients with a history of hypertension or diabetes, as well as rates of tobacco and alcohol use, showed progressive increases. Concurrently, patients in higher tertiles exhibited significantly elevated levels of BMI, FMR, PLT, ALT, UA, TC, TG, LDL-C SBP, and DBP, while demonstrating a lower proportion of elderly individuals and reduced HDL-C levels. The prevalence of NAFLD was also markedly higher in the higher UHR tertiles. However, no significant differences were observed in AST or FBG levels across the tertile groups.

**Table 1:** Baseline characteristics of the study population by tertiles of UHR.

Variables	Tertile 1 (*n* = 530)	Tertile 2 (*n* = 532)	Tertile 3 (*n* = 530)	*p*
Male (*n*, %)				**<0.001**
Female	302 (56.98)	103 (19.36)	39 (7.36)	
Male	228 (43.02)	429 (80.64)	491 (92.64)	
Age≥60 years (%)	195 (36.79)	182 (34.21)	152 (28.68)	**0.016**
Tobacco use (%)				**<0.001**
No	439 (82.83)	325 (61.09)	290 (54.72)	
Yes	91 (17.17)	207 (38.91)	240 (45.28)	
Alcohol use (%)				**<0.001**
No	433 (81.70)	331 (62.22)	308 (58.11)	
Yes	97 (18.30)	201 (37.78)	222 (41.89)	
Hypertension (%)				**<0.001**
No	273 (51.51)	207 (38.91)	169 (31.89)	
Yes	257 (48.49)	325 (61.09)	361 (68.11)	
Diabetes (%)				**<0.001**
No	410 (77.36)	348 (65.41)	336 (63.40)	
Yes	120 (22.64)	184 (34.59)	194 (36.60)	
BMI (kg/m^2^)	24.26 ± 2.93	25.45 ± 2.60	26.51 ± 2.79	**<0.001**
FMR	0.42 ± 0.13	0.38 ± 0.11	0.37 ± 0.10	**<0.001**
PLT (109/L)	215.42 ± 52.72	211.45 ± 55.52	206.58 ± 51.32	**0.026**
ALT (U/L)	21.82 ± 18.21	26.85 ± 19.23	30.02 ± 20.92	**<0.001**
AST (U/L)	22.51 ± 11.29	23.04 ± 11.19	23.84 ± 10.39	0.140
UA (umol/L)	283.88 ± 59.72	363.94 ± 59.37	452.44 ± 79.17	**<0.001**
FBG (mmol/L)	5.44 ± 1.79	5.60 ± 1.65	5.59 ± 1.50	0.212
TC (mmol/L)	4.63 ± 1.02	4.40 ± 1.17	4.40 ± 1.06	**<0.001**
TG (mmol/L)	1.26 ± 1.12	1.75 ± 1.23	2.70 ± 2.24	**<0.001**
HDL-C (mmol/L)	1.41 ± 0.31	1.07 ± 0.17	0.87 ± 0.16	**<0.001**
LDL-C (mmol/L)	2.75 ± 0.86	2.67 ± 0.93	2.58 ± 0.88	**0.007**
SBP (mmHg)	129.50 ± 16.74	131.57 ± 16.04	131.74 ± 14.53	**0.037**
DBP (mmHg)	79.72 ± 11.38	82.04 ± 11.03	83.04 ± 10.92	**<0.001**
NAFLD (%)				**<0.001**
No	293 (55.28)	197 (37.03)	129 (24.34)	
Yes	237 (44.72)	335 (62.97)	401 (75.66)	

Data are shown as mean ± standard deviation (SD) or median (IQR) for continuous variables and proportions (%) for categorical variables. UHR, serum uric acid to high-density lipoprotein cholesterol ratio; BMI, body mass index; FMR, fat-to-muscle ratio; PLT, platelet count; ALT, alanine aminotransferase; AST, aspartate aminotransferase; UA, serum uric acid; FBG, fasting blood glucose; TC, total cholesterol; TG, triglycerides; HDL-C, high-density lipoprotein cholesterol; LDL-C, low-density lipoprotein cholesterol; SBP, systolic blood pressure; DBP, diastolic blood pressure; NAFLD, non-alcoholic fatty liver disease. Bold values indicate statistical significance.

### 3.2 Association between UHR and NAFLD

Table 2 presents the association between the UHR and NAFLD. In the unadjusted Model 1, a higher UHR was significantly associated with increased NAFLD risk (OR = 1.87, 95% CI 1.66–2.10, *p* < 0.001). This association persisted after sequential adjustment for confounders in Model 2 (OR = 1.67, 95% CI 1.46–1.91, *p* < 0.001) and Model 3 (OR = 1.22, 95% CI 1.03–1.46, *p* = 0.022). When analyzed as tertiles (categorical), a significant positive trend was observed across increasing UHR tertiles (*p* for trend < 0.001). Compared to the lowest tertile, the unadjusted ORs for NAFLD were 2.10 (95%CI 1.65–2.69, *p* < 0.001) for tertile 2 and 3.84 (95% CI 2.96–4.99, *p* < 0.001) for tertile 3. Upon full adjustment (Model 3), a significant positive trend across tertiles remained (*p* for trend = 0.022). However, when examining individual tertiles, the association was significantly elevated only in the highest tertile (Tertile 3) compared to the lowest (fully adjusted OR = 1.56, 95% CI 1.07–2.27, *p* = 0.022). The point estimate for the middle tertile (Tertile 2) suggested a potential increase in risk (OR = 1.29), but this association was not statistically significant (95% CI 0.94–1.76, *p* = 0.115).

**Table 2:** Association between UHR and NAFLD.

	Model 1		Model 2		Model 3	
	OR (95%CI)	*p*	OR (95%CI)	*p*	OR (95%CI)	*p*
UHR (per 1 SD)	1.87 (1.66–2.10)	**<0.001**	1.67 (1.46–1.91)	**<0.001**	1.22 (1.03–1.46)	**0.022**
UHR (Tertile)						
Tertile 1	Ref		Ref		Ref	
Tertile 2	2.10 (1.65–2.69)	**<0.001**	1.75 (1.34–2.30)	**<0.001**	1.29 (0.94–1.76)	0.115
Tertile 3	3.84 (2.96–4.99)	**<0.001**	3.03 (2.25–4.08)	**<0.001**	1.56 (1.07–2.27)	**0.022**
*p* for trend		**<0.001**		**<0.001**		**0.022**

Model 1: unadjusted. Model 2: sex, age, tobacco use, alcohol use, hypertension and diabetes. Model 3: adjusted for variables in Model 2 plus body mass index, fat-to-muscle ratio, platelet count, alanine aminotransferase, aspartate aminotransferase, fasting blood glucose, total cholesterol, triglycerides, low-density lipoprotein cholesterol, systolic blood pressure and diastolic blood pressure. UHR, serum uric acid to high-density lipoprotein cholesterol ratio; NAFLD, non-alcoholic fatty liver disease; OR, odds ratio; CI, confidence interval. Bold values indicate statistical significance.

**Figure 1:**
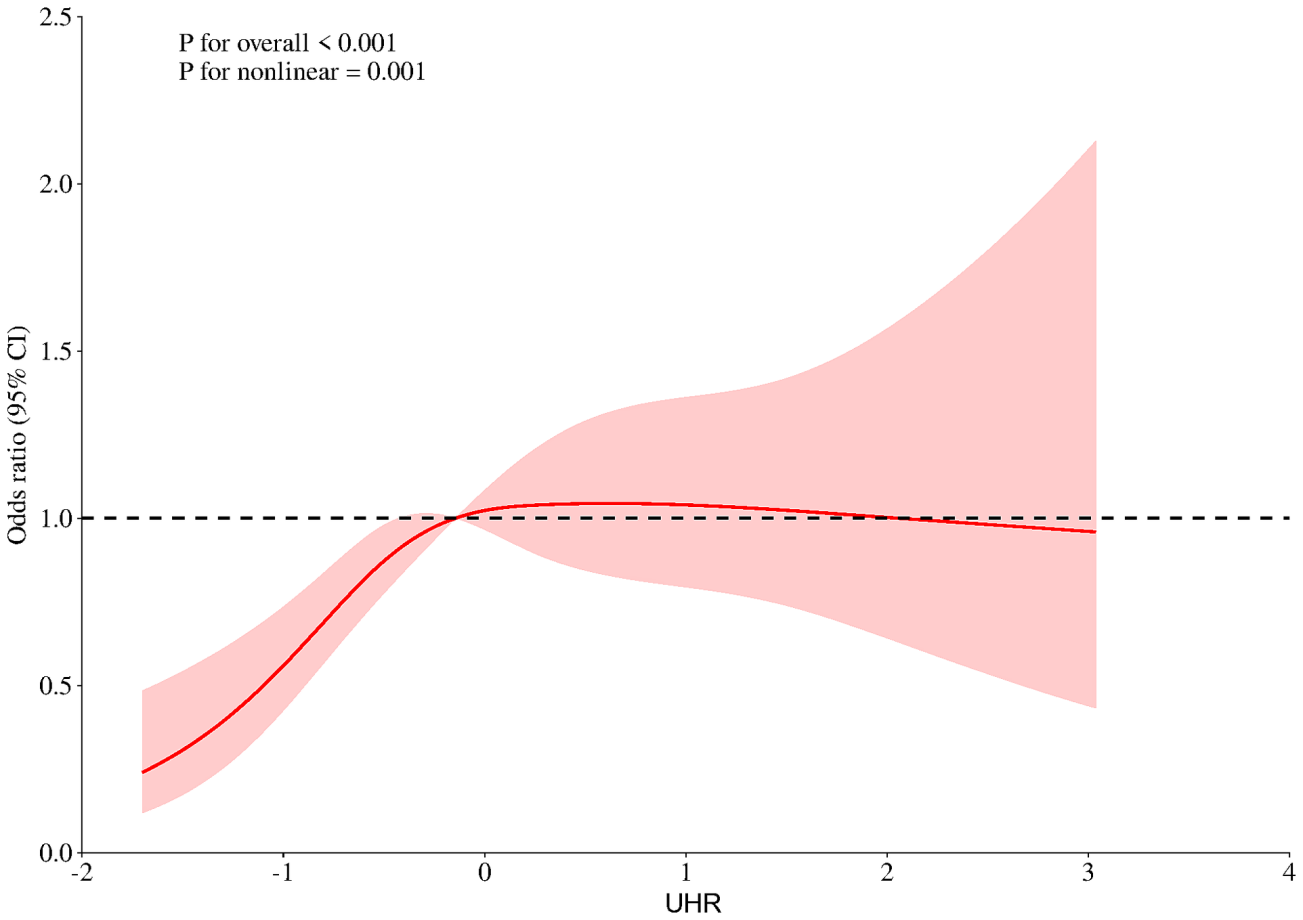
Restricted cubic spline models. Restricted cubic spline models with multivariable-adjusted associations were adopted to demonstrate dose–response associations between UHR and NAFLD. UHR, serum uric acid to high-density lipoprotein cholesterol ratio; NAFLD, non-alcoholic fatty liver disease; OR, odds ratio; CI, confidence interval.

To facilitate cross-study comparability and because the raw UHR distribution was positively skewed, we first standardized UHR into Z-scores (mean = 0, SD = 1). RCS regression then revealed a non-linear dose–response relationship between the standardized UHR and NAFLD (*p* for nonlinear = 0.001; Figure 1). Subsequent segmented regression identified a threshold at Z = −0.75 (likelihood-ratio test *p* < 0.001). Below this cut-off (*Z* < −0.75), each 1-SD increase in UHR was associated with a 4.6-fold higher odds of NAFLD (OR = 4.64, 95% CI 1.57–13.68, *p* = 0.005). At or above the cut-off (Z ≥ −0.75), the UHR was no longer significantly related to NAFLD risk (Table 3).

**Table 3:** Threshold effect analysis of UHR on NAFLD using a two-piecewise logistic regression.

Model	Adjusted OR (95% CI)	*p*
Model 1 Fitting model by standard linear regression	1.22 (1.03–1.46)	**0.022**
Model 2 Fitting model by two-piecewise linear regression		
Inflection point	−0.75	
< − 0.75	4.64 (1.57–13.68)	**0.005**
≥ − 0.75	1.09 (0.89–1.34)	0.390
*p* for likelihood test		**<0.001**

UHR, serum uric acid to high-density lipoprotein cholesterol ratio; NAFLD, non-alcoholic fatty liver disease; OR, odds ratio; CI, confidence interval. Bold values indicate statistical significance.

### 3.3 Subgroup analyses

**Figure 2:**
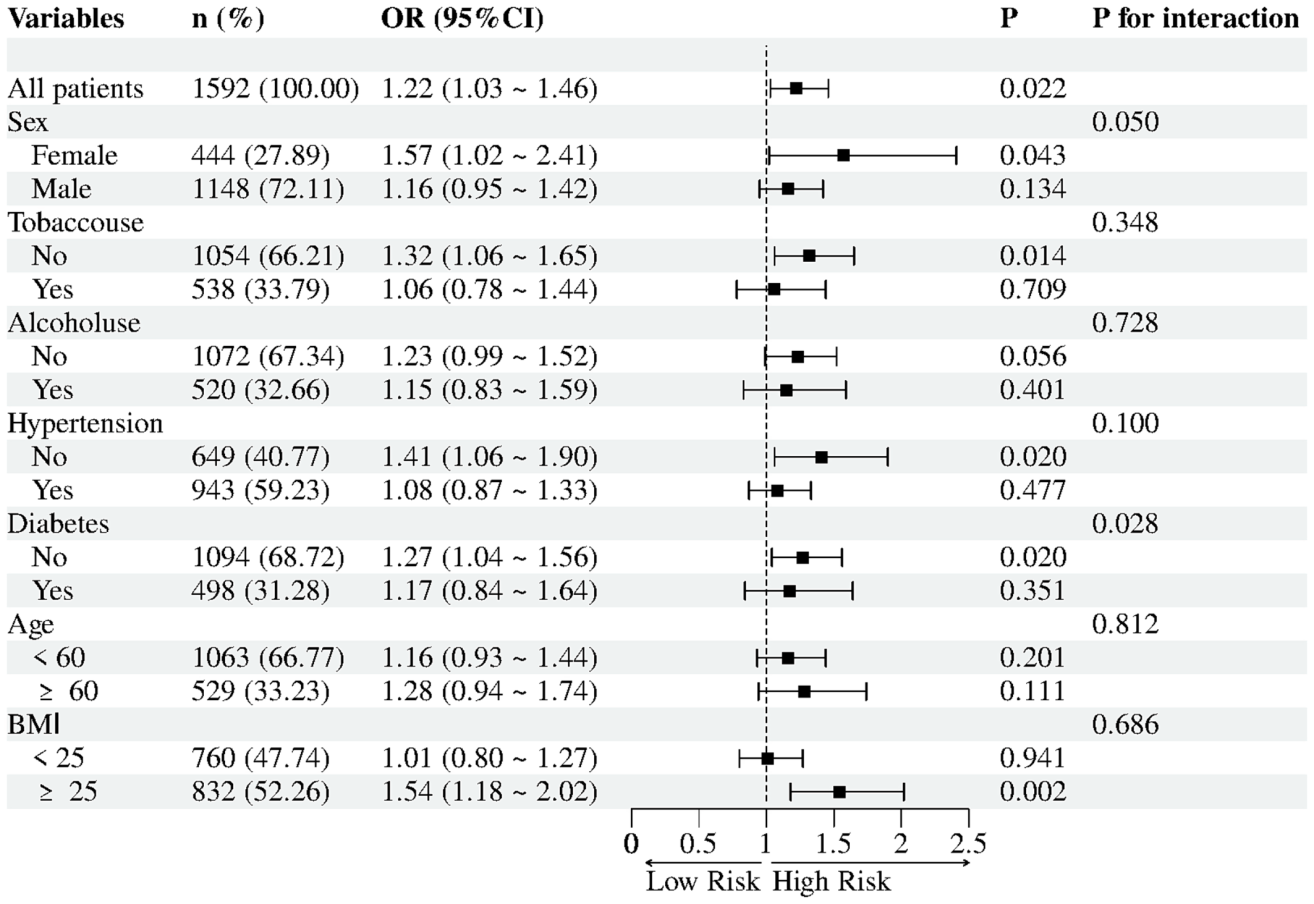
Subgroup analyses for association between UHR (per 1 SD) and NAFLD.

Subgroup analyses evaluated potential effect modification in the association between UHR (per 1 SD increase) and NAFLD risk (Figure 2). Diabetes status significantly modified the association between UHR and NAFLD (P-interaction = 0.028): UHR remained positively associated with NAFLD in non-diabetic participants (OR = 1.27,95% CI 1.04–1.56, *p* = 0.020) but not in diabetic participants (OR = 1.17, 95% CI 0.84–1.64, *p* = 0.351). Sex, tobacco use, hypertension and BMI did not reach statistical interaction (all P-interaction ≥ 0.05); nevertheless, significant positive associations were observed in females (OR = 1.57, 95% CI 1.02–2.41, *p* = 0.043), non-users of tobacco (OR = 1.32, 95% CI 1.06–1.65, *p* = 0.014), non-hypertensive individuals (OR = 1.41, 95% CI 1.06–1.90, *p* = 0.020) and those with BMI ≥ 25 kg/m^2^ (OR = 1.54, 95% CI 1.18–2.02, *p* = 0.002). Conversely, no significant relationships were detected in the corresponding counterpart groups (all *p* > 0.05). Similarly, no association was observed between UHR and NAFLD when participants were stratified by alcohol consumption or age (all *p* > 0.05).

### 3.4 The ability of UHR to predict NAFLD

**Figure 3:**
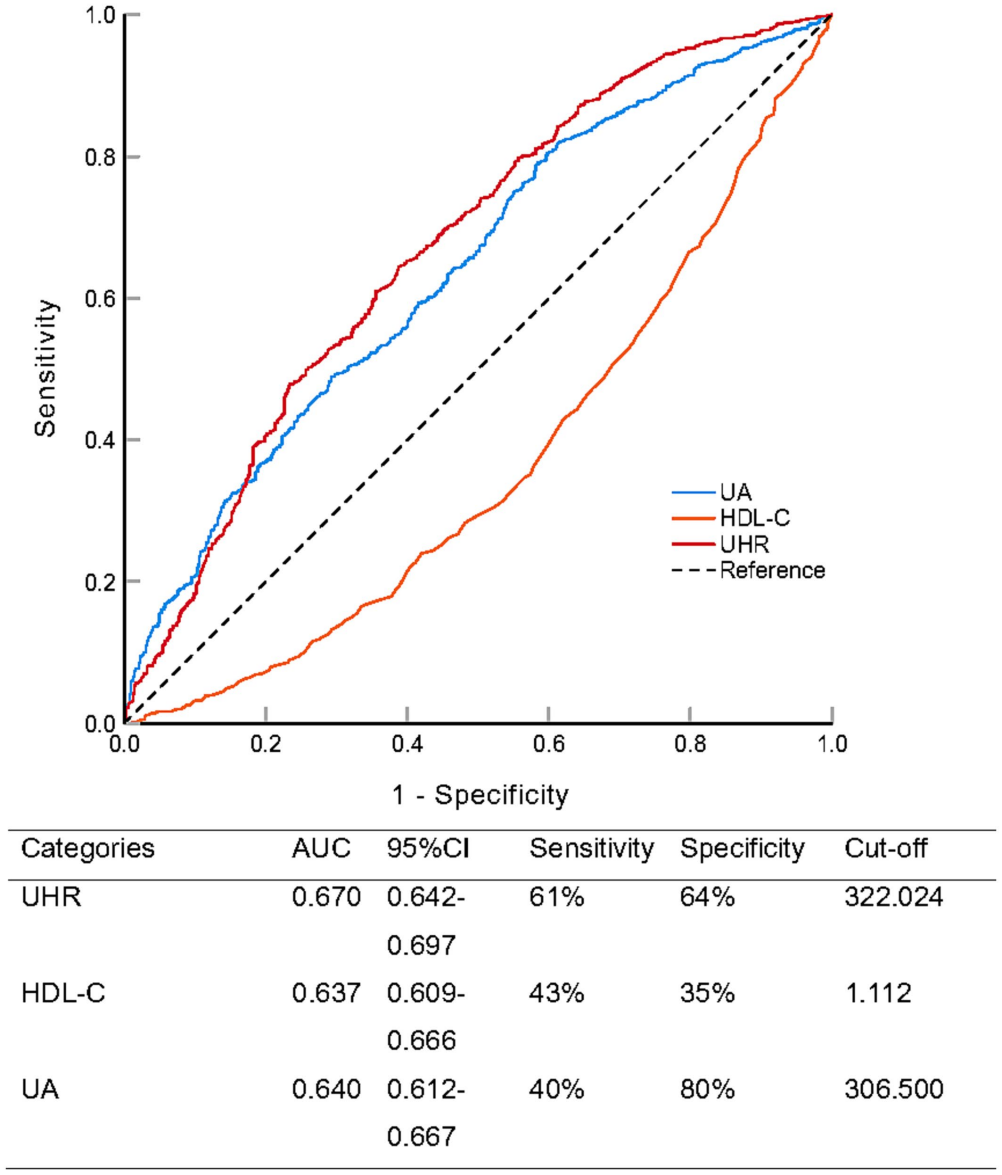
Receiver operating characteristic analysis comparing the predictive ability of serum uric acid to high-density lipoprotein cholesterol ratio (UHR), uric acid (UA), and high-density lipoprotein cholesterol (HDL-C) for non-alcoholic fatty liver disease.

To evaluate the predictive performance of the UHR for NAFLD in the overall population, we performed ROC analysis and compared it with UA and HDL-C (Figure 3; Table 4). The area under the curve (AUC) for UHR in the ROC analysis was 0.670 (95% CI: 0.642–0.697), which was considerably higher than that of UA (AUC = 0.640, 95% CI: 0.612–0.667; *p* = 0.001) and HDL-C (AUC = 0.637, 95% CI: 0.609–0.666; *p* < 0.001). At the optimal UHR cut-off value of 322.024, the sensitivity for predicting NAFLD was 61% and the specificity was 64%. This suggested that UHR may be a more suitable indicator for NAFLD compared to UA or HDL-C alone, although its discriminative ability, according to conventional benchmarks, was still modest and indicative of a fair rather than excellent predictor.

**Table 4:** Paired comparison of ROC curves (DeLong’s test).

Categories	Difference of AUC	Standard error	95% CI	*z*-value	*p*
UHR vs. UA	0.030	0.167	0.012–0.048	3.315	**0.001**
UHR vs. HDL-C	0.307	0.169	0.254–0.360	11.367	**<0.001**

ROC, receiver operating characteristic; AUC, area under curve; CI, confidence interval; UHR, serum uric acid to high-density lipoprotein cholesterol ratio; UA, serum uric acid; HDL-C, high-density lipoprotein cholesterol. Bold values indicate statistical significance.

## 4 Discussion

In this large, single-center, cross-sectional study derived from the Dryad database, we found a robust, positive and non-linear association between the UHR and NAFLD. After multivariable adjustment, each 1-SD increase in UHR remained independently associated with 22% higher odds of NAFLD (OR = 1.22). The nonlinear dynamic—characterized by a dramatic 4.6-fold surge in NAFLD odds per SD increase below the Z-score threshold of −0.75, contrasted by nonsignificant effects above this cutoff—suggested a pathophysiological tipping point in UHR-associated hepatic steatosis. ROC analysis showed that UHR (AUC = 0.670) outperformed both UA and HDL-C alone, although absolute discriminative ability was modest. Subgroup analyses further underscored the heterogeneity of the association between UA and HDL-C. Collectively, these data suggested that UHR may serve as an inexpensive, readily available biomarker to flag individuals at heightened NAFLD risk.

Recent evidence from diverse populations has consistently identified UHR as a robust correlate of NAFLD (11–16). In a small cross-sectional study conducted in Turkey, Kosekli et al. suggested that the UHR could be used as a practical marker for identifying hepatic steatosis (13). In a cross-sectional analysis of 3,766 American adult, each 1-SD increment in the UHR was associated with a 33.1% increase in the odds of NAFLD (14). Similarly, a Chinese case–control study reported an even stronger gradient: individuals in the highest UHR quartile exhibited an adjusted odds ratio of 3.888 (95% CI 2.324–6.504) for ultrasonographically diagnosed NAFLD after controlling for age, sex, BMI and metabolic covariates (12). Extending these observations, three independent cohort investigations conducted exclusively in non-obese Chinese populations convergently demonstrated that elevated UHR remains an independent predictor of incident or prevalent NAFLD (11, 15, 16). Our study not only corroborates these prior findings in an independent Chinese population, but also extends them by (i) rigorously controlling for a wider array of confounders, (ii) uncovering a threshold effect, and (iii) demonstrating that diabetes status significantly modifies the UHR-NAFLD relationship.

UA and HDL-C mirror opposite sides of the metabolic disturbance that characterizes NAFLD. UA is the final product of purine metabolism; when its concentration rises, the risk of NAFLD increases and it becomes an independent driver of both disease onset and liver-injury progression (20, 21). This risk appears to be mediated by several interlocking mechanisms: UA provokes inflammation, boosts NADPH oxidase subunit-4 (NOX4) dependent lipogenesis, generates reactive oxygen species, activates the NOD-like receptor family pyrin domain containing 3 (NLRP3) inflammasome, and triggers endoplasmic-reticulum stress through sterol regulatory element-binding protein 1 (SREBP-1) (8). HDL-C, by contrast, is produced mainly in the liver (9), and its levels fall with physical inactivity, smoking, obesity, and diabetes—the very factors that predispose to NAFLD (22–24). Consequently, NAFLD patients commonly have lower HDL-C concentrations than individuals without the disease (25). HDL-C itself is anti-atherogenic: it curbs monocyte recruitment, suppresses pro-inflammatory cytokines and adhesion molecules, and exerts both antioxidant and anti-inflammatory actions (9, 10). High UA levels increased insulin resistance, and insulin resistance could reduce the concentration of HDL-C. High UA fosters insulin resistance (26), which in turn suppresses HDL-C formation (9), creating a vicious circle that is important to NAFLD pathophysiology (27). The UHR therefore distills this entire chain—oxidative stress, loss of defense and insulin resistance—into a single, easily measured number that outperforms UA or HDL-C alone in predicting NAFLD risk. Nevertheless, our threshold analyses indicate that these pathways appear to be activated chiefly once UA has risen disproportionately relative to HDL-C; beyond this tipping point, further increases in UA relative to HDL-C do not translate into additional NAFLD risk.

Our subgroup analyses revealed that the positive association between UHR and NAFLD was confined to participants without diabetes, whereas no relationship was observed in those with established diabetes (P-interaction = 0.028). Several non-mutually exclusive mechanisms may explain this divergence. First, chronic hyperglycemia reprograms both purine and lipoprotein pathways: excess glucose drives uric acid synthesis and impairs renal excretion (28), while it lowers HDL-C concentrations and cripples HDL function through widespread changes in particle proteome and lipidome (29). The resulting, narrowed UHR range markedly attenuates its ability to discriminate NAFLD risk. Second, when absolute or relative insulin deficiency sets in, hyperinsulinemia, advanced glycation end-products, and *β*-cell dysfunction emerge as the principal engines of hepatic fat accumulation (30, 31), effectively relegating the UA–HDL axis to a subordinate role. UHR’s strong predictive power in non-diabetics positions it as a screening tool for early NAFLD in pre-metabolic syndrome populations. Routine UHR assessment could identify “at-risk” individuals before diabetes diagnosis, enabling lifestyle interventions to halt progression.

The clinical contribution of this study was threefold. First, the UHR outperformed its individual components as a diagnostic indicator, providing an immediately applicable risk-stratification tool based on routine laboratory data. Second, we identified a Z-score threshold of −0.75 that singled out a high-risk cohort for intensified monitoring—a cut-off absents from previous UHR research. Third, subgroup analyses demonstrated that UHR retained predictive power specifically among individuals without diabetes, suggesting that it could flag subclinical metabolic derangement long before standard clinical markers surfaced. Consequently, UHR may serve as an early sentinel for NAFLD in seemingly “healthy” populations, creating a window for timely preventive intervention.

Several limitations warrant cautious interpretation. Firstly, the cross-sectional design precludes causal inferences, and prospective cohorts are needed to confirm UHR’s predictive value. Secondly, a male-to-female ratio of 2.59 limits generalizability, particularly because the association was attenuated in females. Thirdly, residual confounding from unmeasured factors cannot be excluded. Most notably, we lacked data on the use of medications that affect uric acid or lipid metabolism (e.g., allopurinol, statins) and on detailed dietary patterns, both of which could influence UHR and NAFLD risk. Fourthly, NAFLD was diagnosed by imaging rather than biopsy, potentially missing early fibrosis or inflammatory sub-phenotypes. Finally, the Z-score-derived threshold demands external validation across diverse ethnicities and age groups before universal adoption.

In conclusion, a higher UHR is independently associated with increased odds of NAFLD in Chinese adults, with evidence of a non-linear dose–response relationship and significant effect modification by diabetes status. Although the absolute predictive increment is modest, the UHR represents a simple, inexpensive biomarker that may complement existing NAFLD risk assessment strategies, especially among non-diabetic individuals.

## Data Availability

The datasets presented in this study can be found in online repositories. The names of the repository/repositories and accession number(s) can be found at: The data that support the findings of this study are openly available in Dryad at https://datadryad.org/dataset/doi:10.5061/dryad.7d7wm3809.
